# Role of Focal Adhesion Kinase in Small-Cell Lung Cancer and Its Potential as a Therapeutic Target

**DOI:** 10.3390/cancers11111683

**Published:** 2019-10-29

**Authors:** Frank Aboubakar Nana, Marie Vanderputten, Sebahat Ocak

**Affiliations:** 1 Institut de Recherche Expérimentale et Clinique (IREC), Pôle de Pneumologie, ORL et Dermatologie (PNEU), Université catholique de Louvain (UCLouvain), 1200 Brussels, Belgium; frank.aboubakar@uclouvain.be (F.A.N.); marie.vanderputten@uclouvain.be (M.V.); 2 Division of Pneumology, Cliniques Universitaires St-Luc, UCL, 1200 Brussels, Belgium; 3 Division of Pneumology, CHU UCL Namur (Godinne Site), UCL, 5530 Yvoir, Belgium

**Keywords:** focal adhesion kinase, small-cell lung cancer, targeted therapy

## Abstract

Small-cell lung cancer (SCLC) represents 15% of all lung cancers and it is clinically the most aggressive type, being characterized by a tendency for early metastasis, with two-thirds of the patients diagnosed with an extensive stage (ES) disease and a five-year overall survival (OS) as low as 5%. There are still no effective targeted therapies in SCLC despite improved understanding of the molecular steps leading to SCLC development and progression these last years. After four decades, the only modest improvement in OS of patients suffering from ES-SCLC has recently been shown in a trial combining atezolizumab, an anti-PD-L1 immune checkpoint inhibitor, with carboplatin and etoposide, chemotherapy agents. This highlights the need to pursue research efforts in this field. Focal adhesion kinase (FAK) is a non-receptor protein tyrosine kinase that is overexpressed and activated in several cancers, including SCLC, and contributing to cancer progression and metastasis through its important role in cell proliferation, survival, adhesion, spreading, migration, and invasion. FAK also plays a role in tumor immune evasion, epithelial-mesenchymal transition, DNA damage repair, radioresistance, and regulation of cancer stem cells. FAK is of particular interest in SCLC, being known for its aggressiveness. The inhibition of FAK in SCLC cell lines demonstrated significative decrease in cell proliferation, invasion, and migration, and induced cell cycle arrest and apoptosis. In this review, we will focus on the role of FAK in cancer cells and their microenvironment, and its potential as a therapeutic target in SCLC.

## 1. Introduction

Lung cancer, which arises from lung epithelial cells, is histologically divided into two main types: small-cell lung cancer (SCLC) and non-small cell lung cancer (NSCLC), which represent 15% and 85% of the cases, respectively [1]. As opposed to SCLC, oncogenic drivers with sensitivity to targeted therapies have been discovered in NSCLC. Tyrosine kinase inhibitors (TKIs) targeting epidermal growth factor receptor (EGFR) mutations, anaplastic lymphoma kinase (ALK) rearrangements, or other oncogenic abnormalities have brought remarkable improvements in the outcome of oncogenic-driven NSCLC patients [2]. Immunotherapy with anti-programmed death-(ligand) 1 (PD-(L)1) immune checkpoint inhibitors (ICIs) has also significantly improved the survival of NSCLC patients without oncogenic drivers [3,4,5,6,7,8,9]. Clinically, SCLC is the most aggressive type of lung cancer, being characterized by a high growth rate, a fast doubling time, and a tendency for early metastasis, with two-thirds of the patients diagnosed with an extensive stage (ES) disease [10,11]. While a good initial response to chemotherapy and/or radiation therapy is observed in most patients, they typically recur or progress rapidly after the primary treatment, with a median overall survival (OS) of 24–38 months in limited stage (LS) [12,13] and 7–10 months in ES [14], and a five-year OS as low as 5% [1]. 

Despite improvements in the understanding of the molecular steps that lead to SCLC development and progression these last years, there are still no effective targeted therapies in SCLC. Rovalpituzumab tesirine (Rova-T) is an antibody-drug conjugate (pyrrolobenzodiazepine (PBD)-dimer cytotoxic) that is directed against Delta-like 3 (DLL3), an inhibitory NOTCH ligand, which has been shown to be overexpressed on the surface of SCLC cells [15]. Despite encouraging preclinical and early clinical results, targeted therapy with Rova-T underperformed in the phase II TRINITY trial, including pretreated SCLC patients with high levels of DLL3 on tumor cell surface [15,16]. After four decades, the only modest improvement in the OS of patients suffering from ES-SCLC has recently been shown in a trial combining atezolizumab, an anti-PD-L1 ICI, with carboplatin and etoposide, chemotherapy agents [17]. In this trial, the OS was 10.3 months in the chemotherapy alone arm, while it was 12.3 months in the chemotherapy plus immunotherapy arm. Based on this positive trial, atezolizumab that is associated to carboplatin an etoposide recently became the new standard of care in the first-line treatment of ES-SCLC [17]. At relapse or progression after a first-line treatment, a rechallenge with platinum and etoposide is proposed to tumors that are considered to be sensitive to platinum (relapse or progression within 60 or 90 days of completion of chemotherapy) [18], while a second-line chemotherapy with topotecan is proposed to tumors platinum-refractory (relapse or progression before three to six months). However, the response rates are poor and OS ranges from 1.2 months to 7.6 months based on systematic reviews of real-world data 15 [19]. These disappointing results highlight the need for novel therapies.

**Figure 1:**
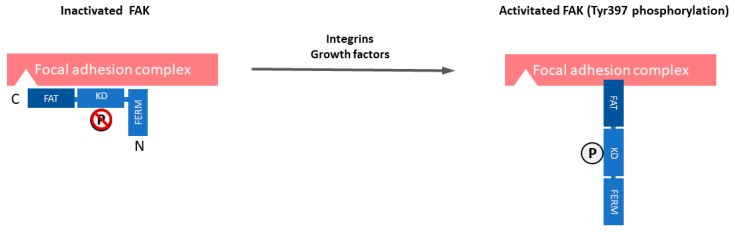
The domain organization and activation of focal adhesion kinase (FAK). FAK is composed of a central kinase domain (KD), an amino-terminal side that is flanked by a protein band 4.1-ezrin-radixin-moesin (FERM) homology domain, and a carboxy-terminal focal adhesion targeting (FAT) domain flanked by proline-rich regions (PRRs). FAK localizes to focal adhesions and is triggered off by extracellular signals such as integrin-mediated adhesion and some growth factors. FAK is maintained in an inactive state by the binding of the FERM domain to the kinase domain, which blocks access to the catalytic site and sequesters the activation loop, as well as the key autophosphorylation site tyrosine 397 (Tyr397). Engagement of integrins with the extracellular matrix (ECM) or growth factors leads to signals that displace the FERM domain, resulting in rapid autophosphorylation of Tyr397, which is a critical event in signal transduction by FAK.

Focal adhesion kinase (FAK) is a 125 kDa non-receptor protein tyrosine kinase that is known to be overexpressed and activated in several cancers, including SCLC [20,21,22,23,24,25,26,27,28]. Unlike receptor tyrosine kinases (RTKs), such as epidermal growth factor receptor (EGFR), non-RTKs, such as FAK, are cytoplasmic enzymes that lack transmembrane and extracellular domains [29]. FAK localizes to focal adhesions and it is triggered off by extracellular signals, such as integrin-mediated adhesion and some growth factors [30]. Therefore, FAK plays a central role in the interaction between cells, including cancer cells and their microenvironment. The FAK structure includes an NH2-terminal Protein4.1-ezrin-radixin-moesin (FERM) domain, a central kinase domain, two proline-rich motifs, and a COOH-terminal focal adhesion targeting (FAT) domain. FAK is maintained in an inactive state by the binding of the FERM domain to the kinase domain, which blocks access to the catalytic site and sequesters the activation loop, as well as the key autophosphorylation site tyrosine 397 (Tyr397) (Figure 1). The engagement of integrins with the extracellular matrix (ECM) or growth factors leads to signals that displace the FERM domain, resulting in rapid autophosphorylation of Tyr397, which is a critical event in signal transduction by FAK [30,31]. Tyr397 phosphorylation provides a binding site that recruits and activates Src through the SH2 domains of Src family kinases. The FAK-Src complex therefore maintains Src and FAK in their activated states, creating a functional kinase complex [32]. 

Based on FAK overexpression and/or increased activity in cancer and its known function in multiple biological processes that play a role in the development and progression of cancers, such as crosstalk between cell and his microenvironment, cell growth, survival, adhesion, spreading, migration, invasion, angiogenesis, DNA damage repair, radioresistance, and regulation of cancer stem cells, it has been suggested that increased the expression and/or activity of FAK may have a critical role in cancer development and progression [33]. Therefore, FAK is a potential target for anti-cancer therapy, especially in SCLC, being known to be a highly invasive cancer. Small-molecule inhibitors targeting the FAK kinase domain and preventing FAK activation (Tyr397 autophosphorylation) have been developed. Phase I trials with GSK2256098 [34,35,36], VS-6062 [37], defactinib (VS-6063) [38,39,40], or BI853520 [41,42,43] have shown an acceptable safety profile and favorable pharmacokinetics. Most frequent treatment-related adverse events included digestive disorders (nausea, diarrhea, vomiting), headaches, reversible proteinuria, and unconjugated hyperbilirubinemia [34,35,36,37,38,39,40,41,42]. With GSK2256098, the best response of stable disease was observed in 37% of glioblastoma (three patients, median PFS 5, seven weeks) [36] and in 45% of advanced solid cancers (28 patients) [35]. With VS-6062, 34% of patients (31 patients) with advanced solid tumors exhibited stable disease at six weeks, including one case of SCLC for ≥6 cycles cycles [37]. VS-6063 led to the stabilization of advanced solid tumors in 43% of Caucasian patients (six cases) after six weeks of treatment [38] and in 33% of Asian patients (three cases) during more than 24 weeks (median PFS of 63 days) [40]. Recently, the combination of the FAK inhibitor GSK2256098 and the MEK inhibitor trametinib in recurrent advanced pancreatic ductal adenocarcinoma did not provide significant clinical activity in a phase II trial (PFS of 1.6 month and OS of 3.6 months) [44]. In malignant pleural mesothelioma, defactinib in maintenance after first-line chemotherapy in a phase II trial did not provide any benefit either (PFS of 4.1 months with defactinib vs 4.0 months with placebo, and OS of 12.7 months with defactinib vs. 13.6 months with placebo) [45]. Preoperative administration of defactinib in the ongoing phase II clinical trial NCT02004028 appears promising, with therapeutic activity (13% objective partial response, 67% stable disease, 17% tumor progression) and beneficial modification of the tumoral microenvironment [46]. Several clinical trials with defactinib associated with immunotherapy (NCT02758587, NCT03727880, NCT02943317), RAK/MEK inhibitor (NCT03875820), or chemotherapy (NCT02546531) are ongoing, with some of them being open to SCLC inclusion (Table 1) [34,35,36,37,39,40,41,42,43,44,45,47,48,49,50,51,52,53,54,55,56,57,58,59,60,61]. Other small-molecules targeting the protein-protein interactions between FAK and other proteins, such as VEGFR-3, called scaffolding inhibitors, have been developed and shown to induce antitumoral effects in preclinical studies. Further research is needed to find predictive biomarkers of response to FAK TKI alone or, probably more promising, in association with another drug.

In this review, we will focus on the role of FAK in tumor development and progression and its potential as a therapeutic target in SCLC.

## 2. FAK Overexpression and/or Activation in Human Cancers, Its Frequency and Mechanisms

Increased FAK expression or activity has been observed by various methods (Western blot, IHC, Northern blot, quantitative real-time polymeric chain reaction, immunohistochemistry (IHC)) in many human cancers, including lung, head and neck, oral cavity, thyroid, breast, ovarian, prostate, colon, liver, stomach, pancreas, kidney, skin, and bone cancers [63,64,65,66]. Increased FAK expression or activity has also been reported in various tumor-derived cancer cell lines [64]. 

IHC in 85 human SCLC tissues revealed that total FAK was localized to the cytoplasm of 78/85 (92%) SCLCs, and that its expression was low in 11 (13%), moderate in 17 (20%), and high in 50 (59%) SCLCs [24]. In a more recent study, multiplex immunofluorescence staining in 105 SCLC and 95 non-NSCLC patients, as well as 37 healthy donors, revealed that FAK and phospho-FAK (Y397) expression was significantly higher in lung cancer than in normal lung, and significantly higher in SCLC when compared to NSCLC tissues (*p* < 0.01). Moreover, the ratio between phospho-FAK and FAK staining scores was significantly higher in SCLC than in NSCLC tissues (*p* < 0.01) [67]. In the SCLC cell lines, FAK and phospho-FAK (Y397) expression has also been shown to be increased [28,68].

**Figure 2:**
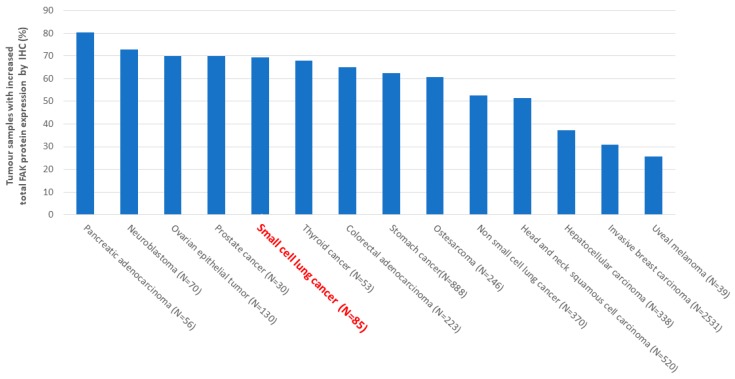
Frequency of focal adhesion kinase (FAK) overexpression at protein level in human solid cancers. A Pubmed search of studies evaluating FAK protein expression in human cancers by immunohistochemistry (IHC) was performed to determine the percentage of cancer samples with increased FAK protein expression. The following keywords were used in the search strategy: FAK [All Fields] AND (“neoplasms” [MeSH Terms] OR “neoplasms” [All Fields] OR “cancer” [All Fields]) AND (“immunohistochemistry” [MeSH Terms] OR “immunohistochemistry” [All Fields]). The results were limited to English language studies. Manual searches of reference articles from applicable studies were performed to identify articles that may have been missed by the computer-assisted search. Abstracts were excluded for cell lines, pre-invasive tumors, if insufficient data to evaluate the methodological quality, absence of tumor total FAK staining, absence of FAK quantification or proportion, absence of proportion of samples overexpressing FAK. Non-eligible trials included ecological studies, case reports, reviews, editorials, and animal trials. This work was conducted in accordance with the PRISMA guidelines (Figure A1). N = number of cancers analysed.

**Figure A1:**
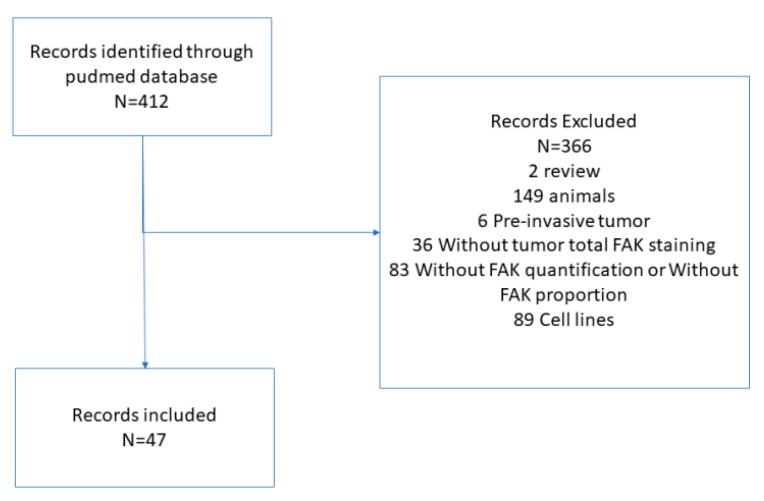
PRISMA guidelines for methodological review of literature related to frequency of focal adhesion kinase (FAK) overexpression at protein level in human solid cancers.

We performed a Pubmed search of studies evaluating FAK protein expression in human cancers by IHC to determine the percentage of cancer samples with increased FAK protein expression. The used methods are described in the legend of Figure 2 and Figure A1. Based on this Pubmed search, we found an overexpression of FAK at the protein level, as evaluated by IHC, in 80% of pancreatic adenocarcinoma, 72% of neuroblastoma, 70% of ovarian epithelial tumors, and many other cancers, including 52% of NSCLC and 69% of SCLC (Figure 2) [20,21,24,26,69,70,71,72,73,74,75,76,77,78,79,80,81,82,83,84,85,86,87,88,89,90,91,92,93,94,95,96,97,98,99,100,101,102,103,104,105,106,107,108,109]. 

**Figure 3:**
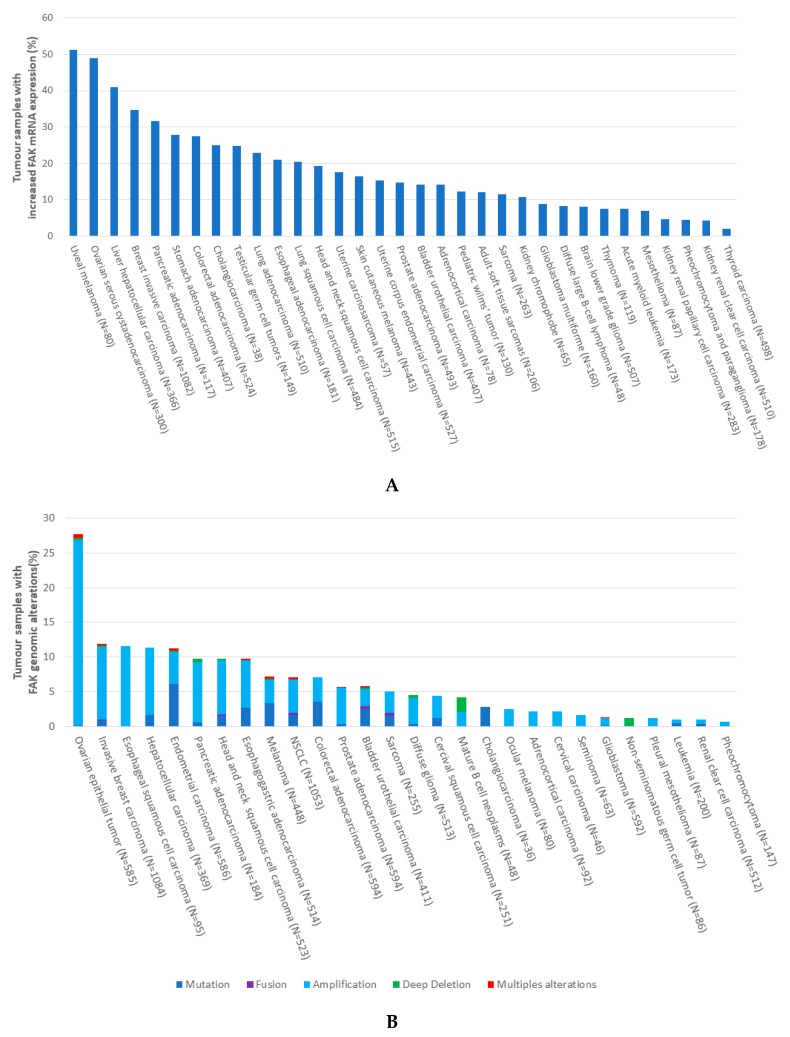
(**A**) Frequency of increased focal adhesion kinase (FAK) expression at mRNA levels in human cancers. The Cancer Genome Atlas (TCGA) was queried using cbioportal.org to determine the percentage of tumor samples with increased levels of FAK mRNA expression. Search criteria included mRNA expression data (Z-scores for all genes) and tumor datasets with mRNA data. N = number of cancers analysed in the TCGA. (**B**) Frequency of focal adhesion kinase (FAK) genomic alterations in human cancers. The Cancer Genome Atlas (TCGA) was queried using cbioportal.org to determine the percentage of samples with FAK genomic alterations (mutations, fusions, amplifications, deep deletions, multiples alterations) in different cancers. Search criteria included PTK2 (FAK). N = number of cancers analysed in the TCGA.

In The Cancer Genome Atlas (TCGA) database [110], we found increased FAK expression at the mRNA level in several human malignancies, including 51% of uveal melanoma, 49% of ovarian serous cystadenocarcinoma, 41% of liver hepatocellular carcinoma, 34% of breast invasive carcinoma, 23% of lung adenocarcinoma, and 20% of lung squamous cell carcinoma, while not being reported in SCLC (Figure 3A). 

Despite recent progress, the underlying mechanisms of FAK overexpression and activation in cancer, especially in SCLC, remain unclear. The control mechanisms include gene alterations, transcriptional regulation, post-translational modifications, and interaction with proteases, phosphatases, etc. Among gene alterations, *FAK* gene amplification within chromosome 8q24.3 and isochromosome formation has been described in many cancers [90,111]. 

Based on the TCGA database [110], the *FAK* copy number gain is found in 26% of ovarian epithelial tumors, 11.5% of oesophageal squamous cell, 10.4% of invasive breast, 9.7% of hepatocellular carcinoma, and less frequently in other tumors, such as 4.8% of NSCLC (Figure 3B), while there are no data related to SCLC. In SCLC, specifically, the genomic profiling of SCLC tumor samples while using genomic comparative hybridization revealed 70 regions of significant copy number gain and 55 regions of significant copy number loss, among which an enrichment of 11 genes associated with the focal adhesion pathway, including amplified *FAK*, was found [28]. The *FAK* gene copy number gain was confirmed by fluorescent in situ hybridization (FISH) in 80% of the SCLC tissues. *FAK* amplification was also correlated to increased FAK mRNA expression. At the protein level, as evaluated by IHC, FAK was expressed in the cytoplasm of 78/85 (92%) SCLC tissues [24]. In the TCGA database, point mutations with a single-base substitution in *FAK* gene, resulting in amino acid substitutions in FAK protein, are found in 6.1% of endometrial carcinoma, 3.5% of colorectal adenocarcinoma, 3.3% of melanoma, 2.7% of cholangiocarcinoma, and less frequently in other tumors, including NSCLC (Figure 3B), while no data are available in SCLC. Somatic mutations (A1004S point mutations) and splicing variants of *FAK* have been reported in 7.7% of human NSCLC (Figure 3B) [112] and they have been shown to exhibit increased autophosphorylation and increased sensitivity to FAK kinase inhibitors as compared with wild-type FAK in patient-derived xenograft models [112]. 

However, *FAK* gene copy number gains and mutations have not always been correlated with increased FAK expression or activity [28]. Therefore, epigenetic mechanisms may also play a role in increasing FAK expression or activity. Analysis of human *FAK* gene promoter has identified putative binding sites for transcription factors. NFκB [113], Argonaute 2 (Ago2) [114], and Nanog [59] are known to activate *FAK* transcription, while TP53 is a well described repressor of the *FAK* promotor [115]. Though not explored in SCLC, this last mechanism might be of particular interest in SCLC where TP53 is universally inactivated [116]. According to TCGA, concomitant TP53 mutation and FAK amplification/mutation co-occurred in 2% of all cancers. However, these data do not include SCLC samples. The lack of material that is dedicated to research is unfortunately a major obstacle in the study of SCLC.

Finally, FAK activation is induced by the engagement of integrins with the ECM or the binding of extracellular growth factors to their receptors. SCLC is well-known to release growth factors, such as bombesin, gastrin-related peptide (GRP), HGF, VEGF, TGF-β, HGF, and FGF, which have been shown to activate focal adhesion pathways in several cancers [117,118,119,120,121,122,123,124,125]. 

Similarly, it has been demonstrated that bombesin, gastrin, and bradykinin phosphorylated FAK in SCLC cell lines in vitro [126], which suggests autocrine and paracrine regulation.

## 3. FAK Role in Proliferation, Cell Cycle, and Survival 

FAK activation during cell adhesion protects cells from anoikis, a form of apoptosis that is induced by cell detachment from ECM, favouring cancer growth and metastasis [127]. FAK is implicated in several pathways that contribute to cell survival. Phosphorylated FAK at Tyr397 can bind PIK3R2, which leads to the activation of AKT that inhibits apoptosis by regulating various molecules. Among other mechanisms, there is the suppression of apoptosis by FAK through c-JUN kinase activation downstream of CAS [33] and the inhibition of RIP interaction with the death receptor complex [128]. 

FAK also induces cell proliferation through the stimulation of cell cycle progression. One of the mechanisms is the formation of FAK/Src complex that allows for Src to phosphorylate FAK at Tyr925 and mediate its interaction with Grb2, which leads to the activation of the RAS-MAPK signaling pathway [40]. Another mechanism involves the FAK-induced increased expression of cyclin D1 and decreased expression of cycline-dependent kinase (Cdk) inhibitor p21 [129,130,131,132]. Other cell cycle regulators, such as cyclin E, Cdk inhibitor p27, and Skp2, also mediate FAK regulation of cell cycle progression [133,134,135,136]. Moreover, FAK is important for tumor cell-induced remodeling of the tumor matrix, which produces a rigid microenvironment and facilitates cell proliferation [137].

Specifically, in SCLC cell lines, it has been shown that the inhibition of FAK activity with PF-573,228, a FAK TKI, decreased proliferation, DNA synthesis, induced cell-cycle arrest in G2-M phases, and increased apoptosis in the NCI-H82, NCI-H146, NCI-H196, and NCI-H446 SCLC cell lines [138]. Treatment with increasing concentrations of PF-228 (0.1 to 10 µM) dose-dependently decreased the FAK phosphorylation (Tyr397) in these four cell lines, without modifying total FAK expression, and the inhibition of FAK activity with 1 to 10 µM PF-228 significantly decreased their proliferation, also dose-dependently (*p* < 0.001 for all tested concentrations beside 1 µM in NCI-H196), as assessed by a WST-1 assay. Cell cycle analysis showed that PF-228 inhibited progression through cell cycle by significantly reducing the S phase and inducing cell cycle arrest in the G2/M phases in the four cell lines after 24h-treatment, dose-dependently (*p* < 0.001). PF-228 at concentrations of 1 to 5 µM also significantly induced apoptosis in the four cell lines, as demonstrated by a dose-dependent increase of PARP p85 expression by WB after 48h-treatment. This was confirmed by flow cytometry in NCI-H82 and NCI-H446 cell lines, with a significant increase of BrdU-positive and activated Caspase 3-positive cells after 48h-treatment (*p* < 0.001 for all tested concentrations, except 1 µM in NCI-H446 in the Caspase-3 assay). Genetic inhibition of FAK through stable transduction with FAK shRNA and/or FAK-related non-kinase (FRNK), a splice variant lacking the N-terminal and kinase domains of FAK, revealed that the FAK-targeting (FAT) domain (attached to focal adhesion complex, where it inhibits pro-proliferative proteins) was necessary to inhibit proliferation, cell cycle progression, and survival [138]. Indeed, FAK shRNA transduction did not affect these functions, while the restoration of FAT domain by FRNK transduction inhibited proliferation, DNA synthesis, and induced apoptosis in the evaluated SCLC cell lines. Additionally, while FAK shRNA transduction increased the active Rac1 level, FRNK re-expression in cells that were previously transduced with FAK shRNA decreased it. Therefore, this study not only suggested that FAK is important in SCLC biology, but also that targeting its kinase domain might have a therapeutic potential, while targeting its FAT domain might have Rac1-mediated pro-tumoral activity and thus should be avoided. 

## 4. FAK Role in Adhesion, Migration, and Invasion

FAK induces morphological changes in cells, including the formation of podosomes or invadopodia, contributing to cell migration [68,139,140]. Moreover, cancer cells overexpressing FAK are able to invade tissues [141]. FAK overexpression contributes to the metastatic phenotype of cancer cells by promoting cell migration and invasion. 

Cell migration is a complex process that consists of several coordinated events, including protrusion of the leading edge, adhesion of the leading edge to the substrate [142], translocation of the cell body, and release of the trailing edge [143]. Thus, a strict regulation of tension at specific times and in specific areas of the cell is required for cell migration [144,145], where FAK plays an important role by sensing the mechanical forces that are generated in or exerted on cells [146], and modulating cell responses to environmental stimuli. Once activated by integrins, G protein-coupled receptors ligands, or growth factors, FAK is autophosphorylated at Tyr397 and activates proteins, such as Src, p130CAS, paxillin, and PIK3R2 [147], to regulate adhesion turnover at the cell front, a process that is central to migration [147,148,149,150,151]. FAK is indeed required for the organization of the leading edge in migrating cells [152]. The formation of a complex between FAK and Src, leading to the phosphorylation of the adaptor molecule CAS by the FAK/Src complex [153,154,155,156,157], is one of the best characterized downstream signaling pathways that mediate FAK-stimulated cell migration. A second mechanism involves FAK interaction with PIK3 and an adaptor molecule, Grb7 [158,159]. A third mechanism involves the modulation of the assembly and disassembly of actin cytoskeleton through the effect of FAK on the Rho family GTPases. Among the Rho family GTPases, FAK/Src signaling has, in particular, been implicated in regulating the activities of Rac1 and RhoA.

Besides its role in cell migration, FAK promotes invasion in normal and cancer cells by various mechanisms. In one of them, FAK promotes the formation of the Src-CAS-Crk-Dock180 complex, which activates Rac1 and JNK, and leads to increased expression or activity of metalloproteinases 2 (MMP2) and 9 (MMP9) [68]. MMPs are concentrated and activated at actin-rich cell/ECM contacts, termed podosomes or invadopodia, which are distinct from focal adhesion. In another mechanism, FAK cooperates with Src to disrupt the E-cadherin-based intercellular adherens junctions [160], contributing to EMT and, therefore, to the invasive phenotype of metastatic carcinomas through increased cell migration and remodelling of the ECM microenvironment [161,162,163]. In SCLC cell lines, the pharmacologic inhibition of FAK with PF-573,228 decreased cell adhesion [28], as well as migration and invasion [138]. In NCI-H69, NCI-H146, and NCI-H209 SCLC cell lines, PF-573,228 induced a dose-dependent decrease of cell adhesion on laminin, with the effect becoming statistically significant at the concentration of 10 µM (NCI-H69: *p* = 3 × 10^−4^, NCI-H146, and NCI-H209: *p* = 1 × 10^−4^ as compared to DMSO) [28]. Moreover, a wound healing assay combined with time-lapse microscopy showed that PF-573,228 decreased the migration velocity of two SCLC cell lines with an adherent component, from 5 to 2.5 µm/min. in NCI-H196 (*p* = 0.0561) and from 9 to 4 µm/min. in NCI-H446 (*p* = 0.0916)) [68]. Modified Boyden chambers showed that PF-573,228, at a concentration of 3 µM, also inhibited invasion, with the number of invasive cells being able to migrate to the lower side of the insert separating the two Boyden chambers, decreasing from 150 to 50 per field (20× magnification) for NCI-H196 and from 45 to five per field for NCI-H446 [68].

## 5. FAK in Epithelial to Mesenchymal Transition

Through epithelial-to-mesenchymal transition (EMT), cancer cells acquire a more motile phenotype, promoting invasion, metastasis, but also conferring resistance to chemotherapies and targeted therapies. Epithelial cancers undergoing EMT acquire transient mesenchymal features, like Vimentin and N-cadherin, which are associated with the loss of epithelial markers E-cadherin and β-catenin [164]. EMT is correlated with poor outcomes in SCLC [165], such as in many other cancers. Identified mechanisms inducing EMT in SCLC include inactive Notch signaling [166], activated MET receptor signaling through hepatocyte growth factor [165], and activated TGFβ/Akt signaling [167].

While FAK-mediated EMT has not yet been explored in SCLC, its important role has been demonstrated in other cancers and non-malignant cells [168,169,170,171]. Impaired FAK functions lead to a defective mesenchymal phenotype during EMT. Hence, upon TGF β-induced EMT, hepatocyte cell lines transduced with FRNK, a genomic method for inhibiting FAK, underwent an incomplete mesenchymal transition, exhibiting a lack of mesenchymal markers MMP9 and fibronectin and a persistence of membrane-bound E-cadherin [168]. Mammary tumor cells with deficient FAK scaffolding function due to Pro 878/881 mutation also displayed incomplete mesenchymal phenotype with increased E-cadherin and decreased N-cadherin, Vimentin, and fibronectin in a mice model [169]. It was associated with decreased metastasis potential and decreased expression of EMT-inducing gene Snail 1 [169]. A similar reduction of Snail 1 in embryonic FAK-null cells has been associated with the inability to display mesenchymal cell characteristics, while the reexpression of FAK restored mesenchymal phenotype and Snail 1 level through PI3K/Akt signaling [170]. In ovarian cancer, FAK controls EMT by upregulating transcription factor KLF8 via the PI3K/Akt pathway [171]. It has been shown that transcription factors Snail 1 and KLF8 repress E-cadherin expression, promoting EMT in various normal and malignant cells [172,173,174]. The inhibition of FAK by a genetic or a pharmacologic method decreased the EMT features and aggressiveness in colorectal carcinoma cell lines [175,176] and triple negative breast cancer cell lines in vitro [177], but not in NSCLC cell lines in vitro [178].

## 6. FAK-Mediated Angiogenesis and Vascular Permeability

**Figure 4:**
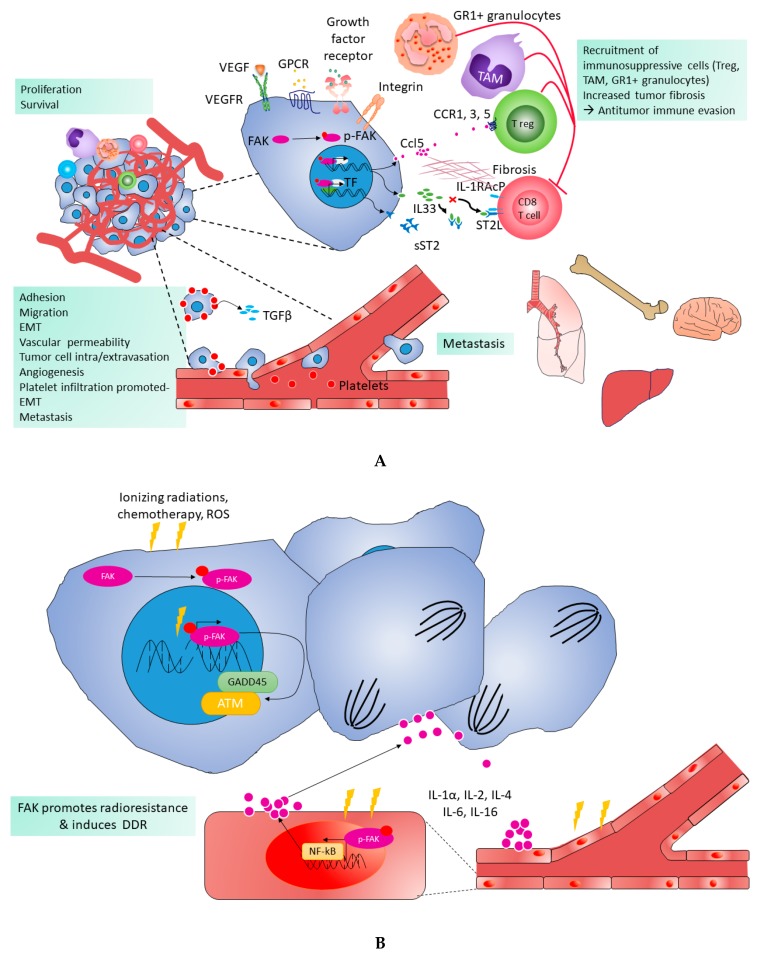
Pro-tumoral functions of FAK. (**A**). FAK is triggered off by integrins, G protein-coupled receptors (GPCR), growth factor receptors, and vascular endothelial growth factor receptor (VEGFR). Activated FAK promotes cell proliferation and survival. FAK also contributes to tumor progression and metastasis via cell adhesion, migration, and promotion of epithelial to mesenchymal transition (EMT). Transient contact between platelets and tumor cells induces TGFβ production by the platelets, which promotes EMT-like transformation and invasive behaviour. In endothelial cell (EC), FAK drives angiogenesis, increases vascular permeability, and regulates platelet extravasation; this facilitates intravasation or extravasation of tumor cells, leading to metastasis. Additionally, FAK induces a tumor protective fibrotic and immunosuppressive tumor microenvironment that promotes antitumor immune evasion. Indeed, FAK induces cytokines (short soluble (sST2), IL33, Ccl5), which lead to the recruitment of immunosuppressive cells, such as regulatory T cells (Treg), tumor-associated macrophages (TAM), and GR1+ granulocytes, as well as to increased tumor fibrosis. Pro-tumoral functions of FAK. (**B**). Ionizing radiations, chemotherapy, and reactive oxygen species (ROS) increase DNA damage and activate FAK in tumor cells. Activated FAK favors the expression of DNA damage repair (DDR) genes such as Growth Arrest and DNA Damage-inducible 45 (GADD45), Ataxia Telangiectasia Mutated (ATM) genes, and Ataxia Telangiectasia and Rad3-related (ATR) genes which play an important role in resistance to drug and radiation. Additionally, in endothelial cells (EC), ionizing radiations activate FAK and NF-kB, which induces the production of various cytokines (IL-1α, IL-2, IL-4 IL-6, IL-16) activating the proliferation of tumor cells. Abbreviations used in the figure and not described in the legend: IL-1RAcP: interleukin-1 receptor accessory protein, ST2L: longer membrane bound form.

The role of angiogenesis and vascular permeability is fundamental to the progression of cancer from localized to advanced-stage disease [179,180,181]. The tumors induce local generation (vasculogenesis) and subsequent growth (angiogenesis) of new vasculature that facilitates the supply of oxygen and nutrients to cancer cells [180]. Moreover, it has been shown that SCLC cells in tumors or in the blood harbours markers of vascular mimicry, including the expression of vascular endothelial cadherin (VE-Cadherin). Therefore, vascular mimicry could supply nutrient and oxygen required for the expansion of SCLC cells [182]. Furthermore, several molecules have been shown to promote angiogenesis and/or vascular permeability, for instance vascular endothelial growth factor (VEGF), hypoxia inducible factor (HIF), fibroblast growth factor (FGF), transforming growth factor beta (TGF-β), hepatocyte growth factor (HGF), tumor necrosis factor-α, angiogenin, ephrins, and angiopoietins [123,179,181,183,184]. SCLC produces many of these pro-angiogenic factors, including VEGF, TGF-β, HGF, and FGF [117,118,119,120,121,122]. Moreover, SCLC displays a higher vascularisation when compared to other tumours. Both high tumor vascularisation and high VEGF expression are associated with a poor outcome in SCLC [122,185]. High VEGF expression has also been correlated to an increased risk of extensive disease [185]. This stressed out the strong connexion between angiogenesis, vascular permeability, and the development of metastases in SCLC, which is a highly metastatic disease with a high prevalence of circulating tumour cells (CTCs) (Figure 4A) [1,11,186,187,188]. Several clinical trials have demonstrated that antiangiogenic agents, such as bevacizumab, pazopanib, and sunitinib, increased the progression-free survival PFS in SCLC, despite that they failed to show a significant benefit in terms of OS [189,190,191,192]. These results are probably related to the absence of relevant biomarkers to select patients that might benefit from antiangiogenic agents.

Interestingly, FAK has a crucial role in angiogenesis and vascular permeability, as demonstrated by the vascular defects in FAK double knockout mice, resulting from the inability of FAK-deficient endothelial cells to organise themselves into vascular networks [193]. Additionally, the overexpression of FAK in vascular endothelial cells promotes angiogenesis [194]. Additionally, VEGF-induced vascular permeability is mediated by FAK signaling (Figure 4A), with the inhibition of FAK activity in endothelial cells suppressing VEGF-stimulated vascular permeability [195]. It has been shown that FAK trigger off by VEGF is abrogated by FAK inhibitors, which decrease vascular permeability and tumor vasculature, preventing tumor growth, metastasis, and immunosuppressive tumor infiltration by cells, such as tumor macrophages and T regulatory cells (Figure 4A) [109,195,196,197,198,199]. Additionally, it has been shown that the withdrawal of antiangiogenic therapy results in accelerated tumor growth and that FAK activation mediates this tumor rebound, which increases angiogenesis and platelet infiltration (Figure 4A) [200]. Interestingly, FAK inhibition prevents tumor rebound after the cessation of antiangiogenic therapy through the inhibition of tumor angiogenesis, platelet-induced tumor cell proliferation, and vascular leakage (Figure 4A) [200,201,202,203]. Of note, there is no data regarding the role of FAK in angiogenesis and vascular permeability, specifically in SCLC.

## 7. FAK and DNA Damage Repair

Exposure to endogenous and exogenous carcinogens (reactive oxygen species, UV light, tobacco smoking, ionizing radiation, platinum chemotherapy…) generates DNA damage in both normal and cancer cells [204]. Signaling pathways that are activated by cells to sense and repair DNA damage, preventing genomic instability, are known as DNA damage repair (DDR) [205,206]. DNA-damaging chemotherapy and radiotherapy are used alone or in combination in the treatment of ES- and LS-SCLC, respectively. SCLC tumors are initially responsive to the treatment, but the development of early resistance limits outcomes. Objective response rates of 80–90% are achieved in LS-SCLC treated by concurrent radiochemotherapy [12,13] and of 60–70% in ES-SCLC treated by platinum-based chemotherapy [207,208], but the median OS is only 25–30 months in LS-SCLC and 12 months in ES-SCLC [12,17,209]. Understanding the underlying mechanisms of acquired or intrinsic radioresistance and/or chemoresistance is important in the improvement of SCLC survival. 

It has been shown that DDR genes and proteins are more highly expressed and activated in SCLC as compared to NSCLC and that blocking these DDR pathways has antitumoral activity in both preclinical [210] and clinical [211] studies, including many different types of cancer. In SCLC specifically, the association of the PARP inhibitor olaparib and the anti-PD-L1 ICI durvalumab in a phase II trial did not meet efficacy criteria, but revealed that responses were only observed in tumors with an inflamed phenotype on tissue biopsies at baseline, which suggests that the tumor microenvironment inflammation phenotype is a potential predictive biomarker [212]. Another phase II trial with the PARP inhibitor veliparib combined or not to the chemotherapy agent temozolomide in recurrent SCLC showed improved overall response rate without improvement of PFS and OS in the combination arm, but patients with SLNF11 (inhibitor of DNA replication)-positive tumors treated with the association had a significantly improved PFS and OS, which suggests that SLNF11 is a predictive biomarker [211]. 

Interestingly, FAK promotes DDR by promoting the transcription of genes favoring DDR, such as growth arrest and DNA damage-inducible 45 (GADD45), ataxia telangiectasia mutated (ATM), and ataxia telangiectasia and Rad3-related (ATR) (Figure 4B) [213,214]. Furthermore, FAK inhibition promotes the hyperactivation of downstream targets of ATM/ATR, such as checkpoint kinase 2 (CHK2) [215]. In in vitro and in vivo preclinical models of NSCLC harbouring KRAS mutations, ionizing radiation leads to FAK activation (Tyr397 phosphorylation), which persists for several hours, while the inhibition of FAK activity leads to an inherent loss of DNA repair capacity and radiosensitizing effects that promote the therapeutic effect of ionizing radiation [213,214,216]. Similarly, FAK has also been shown to regulate human ductal carcinoma in situ (DCIS) cancer stem cells (CSC) activity and response to radiotherapy [217]. While CSC harbor the ability to avoid or efficiently repair DNA damage from radiotherapy and chemotherapy, which play a role in disease recurrence, inhibition of FAK activity potentiated the effect of irradiation in DCIS CSC [217]. Finally, it has been shown that FAK regulates tumor resistance to DNA-damaging therapies through NF-kB activation and subsequent cytokine production. Interestingly, FAK inhibition sensitizes tumour cells to chemotherapy by suppressing NF-kB activation and subsequent cytokine production (IL-1α, IL-2, IL-4, IL-6, IL-16…) (Figure 4B) [217]. Even though no data are available regarding the role of FAK in DDR, specifically in SCLC, we hypothesize that FAK TKI might also be used in SCLC to improve the efficiency of chemotherapy and/or radiotherapy by impairing DDR and/or increasing DNA damage based on these findings in other cancers.

## 8. FAK and Radioresistance

Radiotherapy that is associated with chemotherapy remains the cornerstone of LS-SCLC treatment, despite the frequent emergence of resistance and cancer recurrence. Understanding the underlying mechanisms of acquired or intrinsic radioresistance is important in the improvement of SCLC survival. Several mechanisms have been involved in tumor radioresistance. Among those, adhesion molecules have a key role against radio-induced apoptosis, in a phenomenon called “cell adhesion-mediated resistance” [218,219,220,221,222]. In SCLC, the spontaneous transformation of cell lines in culture, since several months into more adherent and radioresistant sublines highlights this mechanism [223,224]. FAK, as a key player in the focal adhesion pathway, mediates this anti-apoptotic action against ionizing radiation. Hence, the irradiation of a promyelocytic leukemia cell line overexpressing FAK induced less DNA fragmentation and cell death than in the control cells [225]. Accordingly, a proteomic analysis showed that FAK expression was strongly correlated with radioresistance in a large panel of head and neck (HN) squamous cell carcinoma (SCC) cell lines [226]. Moreover, ionizing radiation upregulated the in vitro expression and activation of FAK in breast cancer, glioblastoma, and lung cancer cell lines, leading to acquired radioresistance [227,228]. The inhibition of FAK using genetic (FAK shRNA transduction) or pharmacological (FAK TKI) methods radiosensitized KRAS-mutated NSCLC significantly decreased radiation survival in vitro and in vivo [215]. Similar results have been reported in HNSCC [226,229,230] and in pancreatic carcinoma [218].

Several FAK downstream signaling pathways have been involved in FAK-mediated survival after ionizing radiation. In a promyelocytic leukemia cell line overexpressing FAK, the Phosphoinositide 3-kinase (PI-3K)-Akt survival pathway is constitutively activated. Moreover, FAK prevents radiation-induced cell death by downregulating the mediator of apoptosis Caspase 8 and by upregulating inhibitor-of-apoptosis proteins, like c-IAP and XIAP [225]. Concomitant activation of NF-κB has also been reported [225]. FAK inhibition radiosensitized HNSCC cells lines in vitro through MAPK and Akt signaling dephosphorylation [230]. In spontaneous radioresistant SCLC cell lines, constitutive activation of Akt and MAPK pathways and increased level of active NF-κB are similarly observed [224]. FAK interaction with JNK1 also has an important role for radioresistance in pancreatic carcinoma cell lines [218] and in HNSCC cell lines [218].

Even though not explored in vivo yet, FAK inhibition might be a useful approach for improving radiotherapy efficacy in SCLC. Nevertheless, cautions are mandatory, since the effects of FAK inhibition on radiosensitivity depend on the tumor type. While FAK pharmacological inhibition combined with radiation radiosensitized HNSCC, it did not show any additional effect in vitro on ionizing radiation lethality in non-Kras mutated NSCLC, colorectal carcinoma, and pancreatic carcinoma cell lines [229].

## 9. Regulation of Cancer Stem Cells

CSC hypothesis has been developed over the last 150 years [231] and progressively replaced the clonal evolution theory in carcinogenesis [232]. This model postulates that the tumor arises from a subpopulation of pluripotent cells that are capable of extensive self-renewal and resistance to ionizing radiation and chemotherapies. Altogether, these aggressive subtypes of malignant cells are presumed to be responsible for recurrence after treatment [233]. The existence of CSCs in SCLC has been demonstrated in cell lines and primary tumors [234,235,236,237,238], participating in therapy resistance and the rapid recurrence of SCLC [237,239,240]. CSCs have been identified in SCLC based on the analysis of cell surface markers and functional properties, such as the capacity to exclude Hoechst dye, to form tumorspheres, and to initiate tumor after xenotransplantation in mice, mirroring their tumorigenicity. In SCLC, common markers that are used to study CSCs are CD133, ALDH1, pluripotency-related gene Nanog, Oct3/4, and Sox 2, among others (reviewed in [241]). Some of these markers have been correlated with poor prognosis [242,243,244] 

While not explored yet in SCLC, the critical role of FAK in CSCs maintenance has been described in several cancers. It has been demonstrated that the CSC marker Nanog upregulates FAK, which, in turn, phosphorylates Nanog in CRC cell lines [59]. Upregulation and activation of FAK has also been observed in the presence of Oct 3/4-surexpressing glioblastoma primary cell cultures [245]. CD133, another CSC marker, enhanced cells migration through Src-FAK signaling activation [246]. Furthermore, a strong influence of ECM in sustaining CSCs through FAK signaling has been demonstrated in pancreatic ductal adenocarcinoma, colorectal cancer, and breast cancer [247,248,249]. Additional proof of FAK implication in CSCs is that several drugs that are effective against CSCs act through FAK inhibition [250,251,252,253]. Several studies have demonstrated that FAK inhibition preferentially eliminates CSCs pool in vivo and in vitro in various cancers [217,247,254,255,256,257,258,259]. In pancreatic ductal adenocarcinoma, FAK inhibition with a TKI or shRNA impacted tumor-initiating potential, self-renewal, and metastasis, and improved the response to chemotherapy via CSCs regulation in vitro and in vivo [247]. FAK TKI more efficiently decreased proliferation and survival of the CSCs subpopulation in malignant mesothelioma [254,255], and its administration after chemotherapy improved disease-free survival in a mouse model [255]. In breast cancer, similar effects of FAK inhibition were obtained on the CSCs pool in vivo and in vitro [217,256,257] and on the duration of response after chemotherapy [257]. FAK knockout mice prevented the induction and growth of skin SCC, which suggested the decreased capacity of CSCs generation and maintenance [258]. Finally, colorectal CSCs were preferentially targeted by FAK TKI in vitro in human cell lines as compared to non-CSCs [259]. FAK kinase dependent and independent-functions have both been implicated in CSCs maintenance and regulation in breast cancer [260]. Interestingly, FAK inhibition suppressed β-catenin activation, which confirmed a crosstalk between FAK and Wnt/β-catenin pathway [217,257]. We hypothesize that combination of FAK TKI with conventional treatment might be a pertinent strategy to explore in order to improve outcome given the poor response and rapid recurrence of SCLC after chemotherapy.

## 10. FAK in Tumor Immune Escape

ICIs induced remarkable improvements in tumor response and OS in many types of solid tumors, including NSCLC, both in pretreated and treatment-naive advanced-stage disease [3,4,6,9,261,262]. The most robust objective response rates to ICIs have been shown in tumors with high PD-L1 expression, even though PD-L1 remains an imperfect biomarker [263]. As opposed to NSCLC, there is a lack of correlation between PD-L1 expression and the response to ICIs in SCLC [264] and the efficacy of ICIs in terms of response rates and OS is limited in SCLC patients [17]. The IMpower133 trial, comparing carboplatin plus etoposide with or without atezolizumab, a PD-L1 inhibitor, in the first-line treatment of patients with ES-SCLC, showed only a two-month improvement in OS in the atezolizumab arm. [6]. Nevertheless, it was the first time since several decades that an improved survival was obtained in ES-SCLC. Based on this study, chemotherapy combined with atezolizumab recently became the new standard of care in the first-line treatment of ES-SCLC.

SCLC displays high capacities to escape immune surveillance through several processes. Among those, it has been demonstrated that SCLC cell lines have the capacity to induce regulatory T cell (Tregs) in vitro [265]. This is an important mechanism, as Tregs infiltration in SCLC biopsies has been correlated with the poor survival of patients [265]. Interestingly, a study recently demonstrated a role for FAK in controlling Treg levels in cutaneous and pancreatic tumors [17,266]. In skin SCC, FAK drove the recruitment and expansion of Tregs within the tumor, subsequently impairing the antitumor response of CD8^+^ cytotoxic T lymphocytes [266]. The xenograft of FAK-deficient SCC in mice failed to durably develop and exhibit a CD8+ T cells-dependent tumor regression within 21 days, as opposed to FAK-wild type tumor cells [266]. The pharmacological inhibition of FAK in a skin SCC mouse model decreased the levels of Tregs and increased those of CD8+, which confirmed the key role of FAK in immune escape [266]. Similar results were observed in pancreatic ductal adenocarcinoma and colorectal cancer, where association of FAK inhibitors with immunotherapy markedly improved survival of the mice [17,267]. Mechanistically, FAK controls Tregs infiltration in skin SCC through the transcription of chemokines and cytokines via its nuclear interaction with transcription factors and regulators [199,266]. Among those increased genes, Ccl1, Ccl5, and TGFβ2 have been involved in Tregs conversion and recruitment in various cancers [109,268,269,270,271,272].

Additionally, the immunosuppressive role of myeloid-derived suppressor cells (MDSC) and tumor-associated macrophages (TAM) promoting tumor development by impairing antitumor immunity has been described in various cancers [273]. In SCLC, the peripheric MDSC count has been correlated with poor prognosis [274] and tumor progression induced by TAM has been demonstrated in vitro [275]. Interestingly, FAK TKI also decreased the tumor-infiltrating immunosuppressive cells in pancreatic [17,276] and breast cancers [277]. In SCC, FAK TKI promoted tumor control by reducing tumor-infiltrating regulatory T cells and increasing the T CD8+ T cells [266]. Furthermore, it has been shown that FAK promotes the expression of interleukin-33 (IL-33), soluble secreted form of the IL-33 receptor, called soluble ST2 (sST2), and chemokine CCL5 (CCL5) in SCC cells. Therefore, IL-33 and ST2 mediate FAK kinase-dependent antitumor immune evasion [199].

Even though the role of FAK in immune tumor escape has not been proven yet in SCLC, these studies raise the hope of improving the outcome of patients through the association of FAK TKI with immunotherapy or conventional chemotherapy. In advanced pancreatic cancer, mesothelioma, and NSCLC, a clinical trial evaluating the association of FAK (VS6063) and PD-1 (pembrolizumab) inhibitors is ongoing (NCT02758587).

## 11. Prognostic and Predictive Value of FAK Alterations 

**Figure 5:**
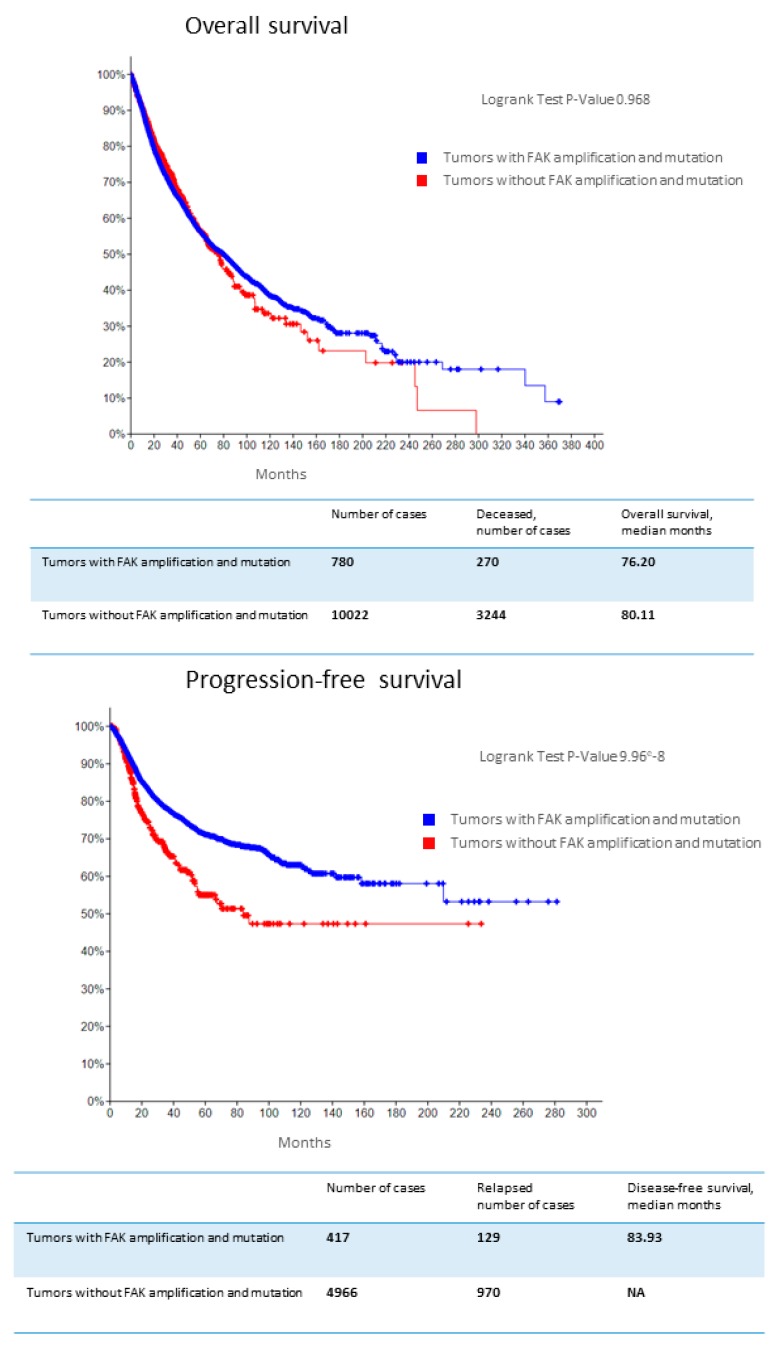
Association of *focal adhesion kinase* (*FAK*) amplification with survival. Kaplan-Meier overall survival and progression-free survival analysis of patients with versus without *FAK* amplification in their tumors (many different cancers included) in The Cancer Genome Atlas (TCGA) database [110].

*FAK* genetic alterations that were reported in the Cancer Cohort of TCGA project were correlated with PFS (Figure 5), and FAK overexpression at mRNA and protein levels were correlated with poor OS in several cancers [200,278]. FAK protein overexpression was associated with worse OS in gastric cancer (HR = 2.646, 95% CI:1.743–4.017, *p* = 0.000), hepatocellular cancer (HR = 1.788, 95% CI: 1.228–2.602, *p* = 0.002), ovarian cancer (HR = 1.815, 95% CI: 1.193–2.762, *p* = 0.005), endometrial cancer (HR = 4.149, 95% CI: 2.832–6.079, *p* = 0.000), gliomas (HR = 2.650, 95% CI: 1.205–5.829, *p* = 0.015), and squamous cell head and neck and digestive cancers (HR = 1.696, 95% CI: 1.030–2.793, *p* = 0.038) [200].

In SCLC, no correlation was found between total FAK expression evaluated by IHC on 85 SCLC tissues and SCLC disease stage, response to therapy, PFS, or OS [24]. Similarly, total FAK and phospho-FAK (Y397) expression evaluated by multiplex immunofluorescence in tissues from 105 SCLC and 95 NSCLC patients did not correlate with PFS or OS [67]. However, a predictive value of response to FAK TKIs cannot be ruled out, even in the absence of a prognostic value. Several clinical trials have evaluated FAK TKI in patients suffering from various advanced-stage cancers, showing antitumor activity (up to 33% objective response rates) and safety [35,36,38,40], while they did not use biomarkers, such as FAK or phospho-FAK expression to identify patients that are likely to respond to FAK TKI. It would be interesting for future clinical trials evaluating FAK TKI to prospectively test total FAK and activated FAK expression as potential predictive biomarkers of response to FAK TKI. 

## 12. Conclusions and Therapeutic Perspectives

In this review, we have presented a brief overview on the role of FAK in cancer development and progression, through its functions in cell growth, survival, adhesion, spreading, migration, invasion, angiogenesis, DNA damage repair, radioresistance, and regulation of CSC. This constitutes the biological rationale to consider FAK as a potential therapeutic target in SCLC. The association of FAK inhibitors with standard therapies of SCLC—platinum-based chemotherapy, radiochemotherapy, or immunotherapy—might have synergistic effects and improve the outcomes of SCLC patients. We hope that the development of specific FAK inhibitors will have clinical translational significance in SCLC by targeting, among others, antitumor immunity, angiogenesis, EMT, regulation of CSC, DDR, and therapy resistance, including radioresistance, which are crucial in SCLC biology.

## Figures and Tables

**Table 1:** FAK inhibitors with anti-tumor activity in preclinical studies and clinical trials.

Name	Type	Specificity	Cancers Targeted	Study Phase	References
TAE-226 Novartis	Kinase inhibitor ATP competitive	FAK, IGF-IR, c-Met, Pyk2	Glioma, ovarian	Preclinical	[47,62]
PF-573,228 Pfizer	Kinase inhibitor ATP competitive	FAK	Prostate, breast	Preclinical	[48]
GSK2256098 GlaxoSmithKline	Kinase inhibitor ATP competitive Reversible	FAK, UGT1A1	Solid tumors (ovarian, pancreatic, meningioma, glioblastoma, malignant pleural mesothelioma)	Clinical: phase I & II	[34,35,36,44,49] NCT00996671, NCT02523014
NVP-TAC544	Kinase inhibitor ATP competitive	FAK	N/A	Preclinical	[50]
VS-4718 (PND-1186) Verastem	Kinase inhibitor ATP competitive Reversible	FAK, Pyk2	Solid tumors (pancreas, breast, ovarian), acute myeloid leukemia, B-cell acute lymphoblastic leukemia	Clinical: phase I	[51]
VS-6062 (PF-562271 and PF271) Verastem	Kinase inhibitor ATP competitive Reversible	FAK, CDK2/CyclinE, CDK3/CyclinE, CDK1/CyclinB, Pyk2	Prostate, pancreatic, head and neck	Clinical: phase I	[37,52]
VS-6063 (Defactinib) Verastem	Kinase inhibitor ATP competitive	FAK, Pyk2	NSCLC, pancreatic cancer, ovarian, malignant pleural mesothelioma, hematologic	Clinical: phase I/Ib & II	[38,39,40,45,53] NCT02758587 NCT02004028 NCT03875820 NCT03727880, NCT02943317, NCT02913716, NCT02465060, NCT02546531
1H-Pyrrolo(2,3-b) Merk Serono	Kinase inhibitor non-ATP competitive	Hinge region of FAK	N/A	Preclinical	[54]
C4 CureFAKtor Pharmaceuticals	Scaffold inhibitor	FAK /VEGFR-3	Neuroblastoma, pancreatic, breast	Preclinical	[55,56,57]
Compound R2 (Roslins) CureFAKtor Pharmaceuticals	Scaffold inhibitor	FAK, p53	Colon, reast	Preclinical	[58]
Y11 CureFAKtor Pharmaceuticals	Scaffold inhibitor	FAK Y397 site	Colon, breast	Preclinical	[59]
BI853520	ATP competitive inhibitor	FAK	Malignant pleural mesothelioma, non-hematologic malignancies	Preclinical, clinical	[42,43,60]

Abbreviations: CDK: Cyclin-dependent kinases 1, 2, 3; FAK: focal adhesion kinase; IGF-IR: insulin-like growth factor 1 (IGF-1) receptor; N/A: data not available; Pyk2: proline-rich tyrosine kinase 2; UGT1A1: UDP-glucuronosyltransferase 1-1; VEGFR-3: vascular endothelial growth factor receptor 3.
